# Comparability of Emotion Dynamics Derived From Ecological Momentary Assessments, Daily Diaries, and the Day Reconstruction Method: Observational Study

**DOI:** 10.2196/19201

**Published:** 2020-09-24

**Authors:** Stefan Schneider, Doerte U Junghaenel, Tania Gutsche, Hio Wa Mak, Arthur A Stone

**Affiliations:** 1 University of Southern California Los Angeles, CA United States

**Keywords:** ecological momentary assessment, daily diaries, day reconstruction method, emotion dynamics, emotion variability, mobile phone

## Abstract

**Background:**

Interest in the measurement of the temporal dynamics of people’s emotional lives has risen substantially in psychological and medical research. Emotions fluctuate and change over time, and measuring the ebb and flow of people’s affective experiences promises enhanced insights into people’s health and functioning. Researchers have used a variety of intensive longitudinal assessment (ILA) methods to create measures of emotion dynamics, including ecological momentary assessments (EMAs), end-of-day (EOD) diaries, and the day reconstruction method (DRM). To date, it is unclear whether they can be used interchangeably or whether ostensibly similar emotion dynamics captured by the methods differ in meaningful ways.

**Objective:**

This study aims to examine the extent to which different ILA methods yield comparable measures of intraindividual emotion dynamics.

**Methods:**

Data from 90 participants aged 50 years or older were collected in a probability-based internet panel, the Understanding America Study, and analyzed. Participants provided positive and negative affect ratings using 3 ILA methods: (1) smartphone-based EMA, administered 6 times per day over 1 week, (2) web-based EOD diaries, administered daily over the same week, and (3) web-based DRM, administered once during that week. We calculated 11 measures of emotion dynamics (addressing mean levels, variability, instability, and inertia separately for positive and negative affect, as well as emotion network density, mixed emotions, and emotional dialecticism) from each ILA method. The analyses examined mean differences and correlations of scores addressing the same emotion dynamic across the ILA methods. We also compared the patterns of intercorrelations among the emotion dynamics and their relationships with health outcomes (general health, pain, and fatigue) across ILA methods.

**Results:**

Emotion dynamics derived from EMAs and EOD diaries demonstrated moderate-to-high correspondence for measures of mean emotion levels (ρ≥0.95), variability (ρ≥0.68), instability (ρ≥0.51), mixed emotions (ρ=0.92), and emotional dialecticism (ρ=0.57), and low correspondence for measures of inertia (ρ≥0.17) and emotion network density (ρ=0.36). DRM-derived measures showed correlations with EMAs and EOD diaries that were high for mean emotion levels and mixed emotions (ρ≥0.74), moderate for variability (ρ=0.38-.054), and low to moderate for other measures (ρ=0.03-0.41). Intercorrelations among the emotion dynamics showed high convergence across EMAs and EOD diaries, and moderate convergence between the DRM and EMAs as well as EOD diaries. Emotion dynamics from all 3 ILA methods produced very similar patterns of relationships with health outcomes.

**Conclusions:**

EMAs and EOD diaries provide corresponding information about individual differences in various emotion dynamics, whereas the DRM provides corresponding information about emotion levels and (to a lesser extent) variability, but not about more complex emotion dynamics. Our results caution researchers against viewing these ILA methods as universally interchangeable.

## Introduction

The use of intensive longitudinal assessments (ILAs) in medical research has risen dramatically over the last few decades. In ILA studies, participants rate their experiences (eg, positive and negative affective states) repeatedly over time, often using electronic data collection via the internet or smartphones. The family of ILA methods encompasses real-time data collection as realized in ecological momentary assessments (EMAs) [1] and day-recall methods, including end-of-day (EOD) diaries [2] and the day reconstruction method (DRM) [3]. ILA methods offer several advantages compared with conventional types of assessment (eg, traditional questionnaires or clinical interviews). By inquiring about experiences that occurred over brief periods (eg, the past few minutes or the last day) in people’s natural environments, ILA methods reduce recall bias and reliance on memory heuristics and can provide self-reports with improved ecological validity. In addition, the fine-grained data resulting from densely repeated assessments can be used to examine short-term, within-person processes that cannot be captured with traditional cross-sectional study designs [4,5].

ILA methods offer many novel insights into people’s health and emotional functioning. For a long time, mental health research has predominantly focused on individuals’ average levels of emotions. However, many aspects of people’s emotional lives are not captured by how they feel on average. ILA adds a needed time dimension that allows assessment of the ebb and flow of subjective experiences and emotions. In many ways, ILA methods encourage a paradigm shift, changing the focus from emotions as static entities to studying them as dynamic processes [6]. For example, many mental health problems, including borderline personality disorder and bipolar disorder, are characterized by emotion dynamics, including increased intrapersonal *variability* or *instability* in affect [7-9]. Relatedly, the degree to which people’s feelings are self-predictive or *linger* over time (described as emotional *inertia* or emotion *network density*) has been viewed as an important indicator of problems with successful emotion regulation [9-11] and as an important feature of mental health. Emotion dynamics may also be associated with physical health. The dynamic interplay of positive and negative affective states (the ability to experience positive and negative affective states in concert, *emotional dialecticism*, or *mixed emotions*) has been proposed as an indicator of emotional complexity and has been shown to benefit health outcomes [12,13].

An important advantage of ILAs is the ability to capture individual differences in emotion dynamics directly from repeated assessments. Researchers have enthusiastically embraced this possibility and have constructed measures of emotion dynamics from a range of ILA data sources, including EMAs, EOD diaries, and the DRM. EMAs have often been regarded as a *gold standard* among ILA methods in that respondents describe their momentary experiences as they are happening in real time (or close to real time) [1]. However, EMAs are relatively expensive to implement and burdensome for participants. EOD diaries, wherein respondents complete a single rating at the end of each day (often using a 24-hour recall period), represent a less costly and more practical alternative that is frequently used for the study of emotion dynamics [9]. The DRM offers yet another alternative that affords a granular assessment of emotional experiences over the course of a day [3,14]. This method has respondents first revive memories of (ie, reinstantiate) the previous day by asking them to divide the day into episodes; for each episode of the day, respondents then provide information about what they were doing and rate their emotional experiences. Compared with EOD diaries and EMAs, the DRM has found less widespread attention in research on emotion dynamics [13]. However, the fact that respondents complete the DRM in a single session (eg, administered over the internet) makes it an attractive method for this purpose because it can be readily implemented with large samples to address population-level research questions [3].

It is often implicitly assumed that data from different ILA methods can be used interchangeably to construct measures of emotion dynamics, even though the specific emotion processes captured by the methods differ in possibly meaningful ways (Table 1). For instance, whereas EMA and the DRM collect information based on relatively brief time intervals between assessments (over periods of hours), EOD diaries provide 1 assessment per day. It is not at all clear that emotion regulation processes occur in similar ways across these different timescales [6]. In addition, whereas EMA protocols typically capture emotion fluctuations that occur both within and across days, EOD diaries target between-day variation, and the DRM is limited to within-day variation (given that the DRM is typically administered only for a single day). The reasons for day-to-day fluctuations in emotions may be conceptually quite different from those generating intraday fluctuations. Furthermore, compared with EMAs, both EOD diary and DRM ratings require retrospection, which can introduce recall bias. Memory heuristics in EOD diary recall (eg, reporting the most salient or peak experiences) have been shown to distort estimates of people’s average experience levels to some extent [15,16], and the assessment of dynamic aspects of emotions may be similarly (or even more) distorted by these memory processes. Another consideration is that a lower number of data points per person negatively impacts the reliability of measures of emotion dynamics [17]; administering the DRM for a single day (ie, the previous day) puts an upper limit on the number of episodes obtained per person, whereas the number of assessments in EMAs and EOD diaries is determined by the research design. In addition, in contrast to EMAs and EOD diaries, the DRM leaves the selection of the number and temporal spacing of episodes to the respondents. This self-selection of episodes may introduce potential selection biases if the experiences respondents choose to report systematically differ from those that are not reported.

**Table 1:** Comparison of ecological momentary assessments, end-of-day diaries, and day reconstruction method measurement features.

Measurement characteristics		Intensive longitudinal assessment methods		
		EMA^a^	EOD^b^ diary	DRM^c^
**Environmental or ecological context**				
	Temporal dynamics covered	Within-day and across multiple days	Across multiple days	Within a single day
	Spacing between targeted experiences	Several minutes to several hours	1 day	Several minutes to several hours
	Timing of completion of assessments	Completed in succession over the course of each day	Completed in succession at the end of each day	All assessments completed in a single session
**Reliance on recall**				
	Targeted reporting period	Moments or very short time periods	Overall summary of all experiences of the day	Summary of experiences during episodes of the day
	Participant recall required	Moment (working memory)	Same day (episodic memory)	Previous day (episodic memory), with reinstantiation
**Potential for selection bias**				
	Selection of targeted experiences	Number and spacing of moments determined by prompting schedule	Fixed (daily)	Number and spacing of episodes determined by respondent
**Reliability of measures of emotion dynamics**				
	Targeted number of measurement occasions	Dependent on prompting schedule (total of 42 in this study)	7 per week (total of 7 in this study)	Approximately 5 to 20 in total, determined by the respondent

^a^EMA: ecological momentary assessment.

^b^EOD: end-of-day.

^c^DRM: day reconstruction method.

Given that research on emotion dynamics derived from ILA is relatively new, researchers are using these measures without solid empirical knowledge about the impact that different ILA methods may have on their findings. To address this problem, this study aims to directly examine the extent to which common ILA methods produce comparable or dissimilar measures of intraindividual emotion dynamics. We used affect ratings collected using EMAs, EOD diaries, and DRM from the same individuals to derive many of the most commonly used measures of emotion dynamics from each method (including mean affect levels; affect variability, stability, and inertia; and emotion network density, emotional dialecticism, and mixed emotions) and examined the convergent validity of these measures across ILA methods. To address the question of whether the use of different ILA methods hampers the reproducibility of findings in health research, we further examined the extent to which measures of emotion dynamics constructed from each of the 3 methods demonstrate corresponding relationships with physical health outcomes (general health, pain, and fatigue). As the relationships between emotion dynamics and physical health are of particular interest in research on later points of the adult life span, this study focused on adults aged 50 years and older.

## Methods

### Participants and Procedures

The data for this study were collected as part of a larger project conducted in the Understanding America Study (UAS). The UAS is a probability-based internet panel maintained at the University of Southern California (USC) Center for Economic and Social Research [18]. It comprises about 8000 panel members, including about 3500 respondents aged 50 years or older. In contrast to convenience (*opt-in*) panels, where people self-select to participate, UAS panel members are recruited through nation-wide, address-based sampling. UAS respondents without previous internet access are equipped with a tablet and broadband internet. In 2017, the UAS started building a data collection environment to enable ILA research with a nationally representative sample [19]. As is typical for large-scale internet panels, UAS panelists answer various surveys on a regular basis, but their level of participation typically does not rise to the magnitude of involvement required for ILAs. The goal of the original study was to demonstrate the feasibility of implementing ILA methods in older participants in this national internet panel. The data for this study were collected between July 2017 and September 2018 during the pilot waves of this project.

The study was approved by the USC Institutional Review Board. UAS panelists aged 50 years or older who were using iOS or Android mobile devices were eligible to participate in the pilot waves. Respondents were screened for current smartphone usage for the purposes of EMA data collection (about 30% were deemed ineligible because they did not use iOS or Android mobile devices). Eligible respondents were provided with information about the project and asked if they were willing to participate. Participants were selected randomly among eligible respondents who consented to participate, with a consent rate of 86% (112/130). As part of the study, participants were asked to complete EMAs and EOD diary questions for 7 consecutive days and to complete the DRM for one of these days (randomly selected). The questions included 1 item addressing positive affect (PA; happiness) and 1 item addressing negative affect (NA; sadness or dejection) that were used for the analyses (additional emotion questions, such as cheerful, frustrated, angry, lonely, or relaxed, were only administered to a small subset of respondents in the DRM and are therefore not used here).

### EMAs

EMA data were collected on participants’ own mobile phones with an app programmed using NubiS software. NubiS is an open-source, secure data collection, storage, and dissemination system developed by the Center of Economic and Social Research at USC. On each study day, respondents were prompted through cell phone beeps to complete EMA questions 6 times per day. Participants could specify the first and last possible prompt time for each day in the app before data collection, where the first prompt could be selected to occur between 6 AM and 11 AM and the last prompt between 7 PM and 11 PM of each day. Prompts were delivered using a stratified random sampling scheme that generated consecutive random prompts within the user-specified time window with a uniform probability between 0.75×(time window/6) and 1.10×(time window/6) hours (approximately between 1 and 3 hours) after the previous prompt. Each time the respondents received the prompt, they had 8 min to start answering questions. A reminder prompt was sent halfway during the 8-min time window if a participant did not immediately respond to an incoming prompt. Respondents were instructed not to respond to any prompt when they were driving and answer questions only if they were in a safe, secure, and private place. Each EMA survey included questions about location, social environment, and physical symptoms that were not analyzed here. The questions examined in this study were “before the prompt, how happy were you feeling” (PA), and “before the prompt, how dejected/blue/downhearted were you feeling” (NA). Responses were given on a 0 to 100 horizontal visual analog scale with anchors that ranged from *not at all* to *extreme*.

### EOD Diaries

Respondents were asked to complete a web-based EOD diary survey in the evening (after 6 PM) of each of the 7 study days. Respondents completed the daily diaries using their laptops or tablets in their UAS account. The EOD diary survey questions paralleled those used in the EMA reports. The PA question was, “please move the slider to represent how happy you felt today,” and the NA question was, “please move the slider to represent how dejected/blue/downhearted you felt today.” Responses were given on a 0 to 100 horizontal visual analog scale with anchors that ranged from *not at all* to *extremely*.

### DRM

The DRM was administered on the web once between days 2 and 7 of the study after participants had completed the daily diary questions. Participants were first asked when they woke up on the previous day and for how many hours they were awake. Next, they were asked to “think of yesterday as a series of scenes in a movie” and to divide the day into episodes. Starting at the time they woke up, participants entered a label (using open-ended text entry) for the first episode that best described what they did during that time, specified an ending time for the episode, and clicked *next* to move to the next episode. This process was repeated for subsequent episodes until participants reached the end of the day. Participants were told that many people define episodes that last between 15 min and 2 hours, but they were encouraged to define as many episodes as made sense to them and could specify episodes of any duration. If participants were awake for more than 24 hours, they were asked to enter episodes for the first 24 hours they were awake. After the complete day had been reconstructed, participants completed questions about where they were, whom they interacted with, and how they felt during each episode. Consistent with the rating scale format used in the original DRM [3], emotions were rated on a 7-point scale from 0=*not at all* to 6=*very much*. Participants were asked to “please rate each feeling on the scale given,” and the emotions presented included *happy* (PA) and *sad* (NA). As the number of scale points used in the DRM differed from the number of scale points used in EMAs and EOD diaries, the DRM scores were transformed into a 0 to 100 scale using the following formula: transformed score=100×(original score+0.5)/7 [20].

### Construction of Measures of Emotion Dynamics

The selection of measures of emotion dynamics was guided by previous reviews of the most commonly employed measures in applied research settings [9,11,21]. All measures were calculated in the same way for each ILA method based on each respondent’s PA and NA ratings across moments (EMAs), days (EOD diaries), or episodes (DRM). To be included in the analyses, we required that a participant provide at least 4 observations for each of the ILA methods, which was deemed the minimum number of observations required to reasonably compute the various measures.

#### Mean PA and NA Levels

Measures of an individual’s mean PA and NA levels represent the most well-known and prominent indicators of psychological well-being. For each ILA method, they were calculated by taking the average of each respondent’s ratings across assessment time points, separately for PA and NA.

#### Variability of PA and NA

Emotion variability captures the magnitude (or amplitude) of fluctuations of a person’s emotional states around the person’s average emotion level [22]. Measures of variability were created by calculating the within-person SD of each respondent’s PA and NA ratings across assessment time points.

#### Instability of PA and NA

Emotional instability measures differ from variability measures in that they explicitly consider the temporal ordering of affective states. Specifically, emotional instability refers to the magnitude of shifts in PA and NA levels across consecutive assessments [7]. The root mean square successive difference measure was used for this purpose, which was calculated by taking the average of the squared differences between successive ratings and taking the square root of this average for each respondent.

#### Inertia of PA and NA

The concept of emotional inertia refers to the degree to which an individual’s emotions are resistant to change, such that levels of PA or NA persistently carry on over time. Inertia is commonly operationalized using the first-order autocorrelation of consecutive measurements in a time series to capture the temporal dependency of a person’s PA and NA ratings [10]. Correspondingly, we obtained measures of inertia in PA and NA as the person-specific autoregressive slope in regression models in which an affect rating at one time point predicts the rating of the same affect item at the subsequent measurement time point.

#### Emotion Network Density

The concept of emotion network density is an extension of the inertia concept to multiple affect items. It evaluates the degree to which multiple emotions predict each other over time, reflecting the extent to which a person’s overall system of PA and NA states is resistant to change. Whereas measures of inertia are calculated separately for each affect item, network density combines the temporal dependencies of multiple affect items in a single measure [23,24]. For 2 emotion items, it comprises the sum of the absolute value of 4 lagged parameters in a vector autoregressive model involving 2 autoregressive and 2 cross-lagged parameters of PA and NA. Specifically, we obtained the person-specific (autoregressive and cross-lagged) parameters of PA and NA (entered simultaneously as within-person centered predictors) in regression models in which either PA or NA served as the outcome variable. The network density measure was then created by taking the absolute value of the 4 regression parameters and calculating the sum of these absolute values separately for each person.

#### Mixed Emotions

The experience of mixed (or bittersweet) emotions has been conceptualized as the extent to which both PA and NA are felt together at the same point in time. The construct of mixed emotions involves the simultaneous activation (or co-occurrence) of both positive and negative experiences [13]. Simply knowing whether an individual has high average levels of PA and NA over time may tell us nothing about whether they experienced a blending of PA and NA at any given point in time [25]. Following previous research, a measure of mixed emotions was calculated using the MIN index [13,25,26]. The index is based on the ambivalence metric proposed by Kaplan [27], which is defined as *total affect* (the sum of ratings for PA and NA) minus *polarity* (the absolute difference between ratings for PA and NA) for a given time point. Arithmetically, this formula is equivalent to taking (2 times) the smaller value of the PA and NA ratings (ie, MIN [PA, NA]) at a given time point [28], such that the MIN index is high only if both emotions co-occur at high levels at that time point. The MIN index was calculated for each time point and averaged across the assessment time points of each respondent.

#### Emotional Dialecticism

The concept of emotional dialecticism (the reverse of the concept of affective bipolarity) refers to the degree to which individuals tend to experience PA and NA independently from each other rather than as bipolar opposites [29,30]. Although conceptually similar to mixed emotions, the measure is calculated as the within-subject correlation between PA and NA ratings of each respondent [29]. As the correlation between PA and NA is expected to be negative on average, more strongly negative values (ie, values approaching a correlation of −1.0) on the measure represent less dialecticism, and less strongly negative values (ie, values approaching or exceeding a correlation of 0) represent more dialecticism.

Several additional considerations regarding the calculation of the measures of emotion dynamics are noteworthy. First, measures that take the temporal ordering into account (ie, measures of emotional instability, inertia, and emotion network density) require that the time intervals between 2 consecutive measurements are approximately equal. For this reason, when calculating these measures from EMA data, time periods from the evening of one day to the morning of the next day were omitted, as were momentary ratings that occurred after a time gap of more than 10 hours. Similarly, consecutive DRM ratings that were more than 10 hours apart from each other (measured from the midpoint of one episode to the midpoint of the next episode) were omitted when calculating these measures. Second, measures of emotion dynamics that involve lagged within-person relationships (ie, measures of inertia and emotion network density) are prone to high imprecision because of sampling error unless the number of measurement occasions is very large [17]. As the number of occasions in this study was relatively modest (especially for daily diaries), we used multilevel models to obtain empirical Bayes estimates of the person-specific regression parameters for calculating the inertia and network density measures. Compared with parameters from regression models that are estimated separately per respondent, the multilevel approach yields more precise parameter estimates for individual respondents [31].

### Health Variables

Self-reported general health, pain, and fatigue were measured at the end of each of the 7 study days as part of the EOD diary assessments. For each health variable, the 7 scores were averaged into a summary measure for each person. Self-reported health was measured with the question “How was your health today?” with response options from the Short Form 36 general health item (excellent, very good, good, fair, or poor) [32]. To assess pain severity and fatigue levels, participants were asked to rate the following statements, *your average level of bodily pain* (no pain at all to extreme pain) and *how fatigued (ie, weary or tired) you were* (no fatigue at all to extreme fatigue), using a 0 to 100 horizontal visual analog scale.

### Statistical Analysis

Data analyses were performed in 3 broad steps. The first step of the analysis examined the extent to which the 3 ILA methods (ie, EMAs, EOD diaries, and DRM) yielded comparable scores for each emotion dynamic. To accomplish this, we compared the means and examined the correlations of measures addressing the same emotion dynamic across the ILA methods. Differences in mean scores were tested using analysis of variance (ANOVA) procedures with an ILA method as a within-person (ie, repeated measures) factor. An omnibus test of overall mean differences between the 3 methods was conducted first, followed by Bonferroni-adjusted pairwise tests of mean differences between the methods. For correlations of emotion dynamic measures between ILA methods, we roughly considered correlations of 0 to 0.35 as low, 0.36 to 0.67 as moderate, 0.68 to 0.89 as high, and 0.90 to 1.00 as indicating very high correspondence, following conventions [33]. To test whether the correlations differed between pairs of ILA methods (such that some ILA methods more highly correspond with each other than with other methods), we used Fisher z-transformed correlation coefficients and conducted Wald tests for differences in dependent correlations as implemented in Mplus version 8.4 (Muthén & Muthén) [34].

Whereas the first analysis step considered each measure of emotion dynamics separately, the second set of analyses examined how the measures are interconnected within each ILA method. Different measures of emotion dynamics may positively or negatively relate with each other in complex ways, and these relationships can be viewed to form a system or network of interdependencies among different features of emotional experience. Thus, evidence that the pattern of associations among the various measures corresponds across ILA methods would support the convergent validity of the methods. To examine this, we first inspected the structure of interconnections graphically using network visualization in the R package qgraph [35]. This technique represents a correlation matrix as a network in which each measure of emotion dynamics is represented as a node, and their interconnections are shown as edges between the nodes, allowing for a visual comparison of the correlation networks between ILA methods. To quantify the similarity of the correlation networks across ILA methods, we capitalized on centrality indices used in network analysis. In centrality analysis, the emotion dynamics are ordered in terms of the degree to which they occupy a central place in the overall network and exhibit many strong associations. We focused on the *simplest centrality metric*, *node strength*, which was calculated for each emotion dynamic as the sum of its absolute correlations with all other emotion dynamics in the network [36]. The ordering of the emotion dynamics’ centralities was then descriptively compared across the ILA methods.

The third step examined the extent to which measures of emotion dynamics constructed from the different ILA methods demonstrate corresponding correlations with the health outcomes (general health, pain, and fatigue). To statistically compare the correlations of each emotion dynamic across the different ILA methods, we conducted Wald tests for differences in dependent Fisher r- to z-transformed correlation coefficients. In addition, effect sizes (Cohen *q*, where values of 0.1, 0.3, and 0.5 can be interpreted as small, medium, and large effects, respectively) were calculated to quantify the magnitude of differences in the correlations between ILA methods. *P* values <.05 were considered significant for all analyses.

## Results

### Descriptive Characteristics

A total of 100 participants completed assessments using all 3 ILA methods, with 10 participants being excluded from the analyses because they provided less than 4 observations for at least one of the ILA methods (1 participant had fewer than 4 observations for all ILA methods, 3 for EMA, and 6 for the DRM), resulting in an analysis sample of 90 participants. The mean age of the analyzed sample was 62.4 years (SD 7.7; range 51-87 years), 57% (51/90) were female, and 63% (57/90) were married. The sample was predominantly White (72/90, 80%) and non-Hispanic (83/90, 92%). The median household income was in the category between US $60,000 and US $74,999, and 57% (51/90) had a college degree. About half (42/90, 47%) were currently working, and about one-third (32/90, 36%) indicated that they were retired. Participants who were excluded from the analyses were less likely to hold a college degree (*P*=.04) but otherwise did not differ from the analysis sample on these demographics.

In terms of participants’ compliance with the ILA protocol, the mean number of completed EMA prompts per person in the analyzed sample was 29.9 (SD 11.2; median 34) out of 42 possible ratings (6 per day across 7 days), yielding an average EMA completion rate of 71.2% (29.9/42). EOD diaries were, on average, completed on 6.9 (SD 0.5; median 7) out of the 7 days, yielding an average completion rate of 99.0% (6.9/7). For the DRM, the mean number of episodes provided per person was 11.8 (SD 5.7; median 11.0), which is roughly comparable with previous research using the DRM [3].

As the expected values of emotion dynamics that involve lagged within-person associations depend on the length of the lag (ie, autocorrelations are expected to be, on average, lower, the longer the lag time is), descriptive statistics for the time distances between ratings for each ILA method were also examined. For EMA prompts, the average distance between assessments (after elimination of overnight gaps and gaps >10 hours) was 2.13 hours (SD 0.89; median 1.94), with an IQR of 1.70 to 2.18 hours. The average time distance between consecutive EOD diaries was 24.01 hours (SD 2.16; median 24.05; IQR 23.32-24.76 hours). For the DRM, the average distance between the midpoints of consecutive episodes was 1.68 hours (SD 1.25; median 1.25; IQR 0.58-2.25 hours).

To evaluate the proportion of the total variance in affect that was accounted for by stable, trait-like differences as opposed to within-person fluctuations, intraclass correlations of PA and NA ratings were calculated for each ILA method, computed as the ratio of between-person variance to total (sum of within- and between-person) variance in ratings. The intraclass correlations were 0.63 (PA) and 0.54 (NA) for EMA reports; 0.57 (PA) and 0.53 (NA) for EOD reports; and 0.60 (PA) and 0.51 (NA) for DRM reports. Thus, between 37% (151.4/403.8) and 43% (175.6/410.9) of the variance in PA was within-person, and between 46% (142.6/311.2) and 49% (121.2/249.2) of the variance in NA was within-person, with consistency across the 3 ILA methods.

### Correspondence of Measures of Emotion Dynamics Across the ILA Methods

ANOVA models yielded no significant differences between the ILA methods for measures capturing mean levels in PA and NA, PA variability, and PA instability, whereas significant method differences were evident for the remaining measures of emotion dynamics (Table 2). In pairwise comparisons between the ILA methods, the averages of measures from EMAs and EOD diaries did not significantly differ from each other, with one exception: the average inertia for NA was smaller for EOD diaries than for EMAs. This difference is consistent with expectations, given a longer lag time between EOD diary assessments compared with the lag times between EMAs. For DRM-derived measures, the averages were less comparable with those from the other ILA methods. The DRM yielded higher averages of inertia for PA and NA, and a higher emotion network density, compared with both EMAs and EOD diaries (consistent with the shorter time lags of the DRM). In addition, the DRM yielded lower average scores for NA variability and NA instability and higher scores for mixed emotions and emotional dialecticism compared with both EMAs and EOD diaries (Table 2).

The correlations between measures derived from EMAs and EOD diaries suggested very high correspondence (ρ>0.90) between these 2 methods for individuals’ mean PA levels, mean NA levels, and mixed emotions, and high correspondence (ρ=0.68 and 0.80) for measures of PA and NA variability (Table 3). EMA- and EOD diary–derived measures further showed moderate-to-high correspondence (ρ ranging between 0.51 and 0.70) for PA and NA instability and emotional dialecticism measures, and low-to-moderate correspondence (ρ ranging between 0.17 and 0.57) for measures of PA and NA inertia and emotion network density. For most of the measures derived from the DRM, the correlations with both EMAs and EOD diaries were significantly weaker by comparison (Table 3). Specifically, the DRM showed high correspondence with EMAs and EOD diaries (ρ ranging between 0.74 and 0.80) for individuals’ mean PA levels, mean NA levels, and mixed emotions, moderate correspondence (ρ between 0.38 and 0.54) for PA and NA variability measures, low-to-moderate correspondence (ρ between 0.19 and 0.41) for PA and NA instability measures, and low correspondence (ρ between 0.03 and 0.32) for the remaining measures (PA and NA inertia, emotion network density, and emotional dialecticism).

**Table 2:** Mean (SD) of measures of emotion dynamics for each assessment method.

Emotion dynamic	Assessment method, mean (SD)			ANOVA^a^ of mean differences	
	EMA^b^	EOD^c^ diary	DRM^d^	*F* value (*df*=2178)	*P* value
PA^e^ mean level	66.24 (16.1)	68.40 (16.1)	66.79 (17.6)	2.61	.08
NA^f^ mean level	12.60 (13.3)	12.12 (14.7)	14.00 (11.6)	2.73	.07
PA variability	11.29 (4.6)	11.65 (6.7)	11.93 (7.8)	0.42	.66
NA variability	9.05^g^ (7.3)	9.02^g^ (9.3)	5.79^h^ (8.5)	10.14	<.001
PA instability	12.92 (6.3)	14.51 (9.1)	13.53 (9.6)	1.16	.32
NA instability	10.41^g^ (8.8)	11.08^g^ (11.6)	6.58^h^ (9.8)	10.13	<.001
PA inertia	0.41^g^ (0.1)	0.38^g^ (0.1)	0.46^h^ (0.2)	8.72	<.001
NA inertia	0.34^g^ (0.1)	0.26^h^ (0.1)	0.42^i^(0.2)	32.49	<.001
Network density	0.86^g^ (0.2)	0.91^g^ (0.2)	0.99^h^ (0.3)	10.23	<.001
Mixed emotions	10.90 (10.6)	10.03^g^ (11.6)	12.42^h^(9.5)	6.24	.002
Dialecticism	−0.34^g^ (0.3)	−0.36^g^ (0.2)	−0.21^h^ (0.2)	13.14	<.001

^a^ANOVA: analysis of variance.

^b^EMA: ecological momentary assessment.

^c^EOD: end-of-day.

^d^DRM: day reconstruction method.

^e^PA: positive affect.

^f^NA: negative affect.

^g,h,i^Means in the same row with different superscripts differ significantly from each other using Bonferroni-adjusted pairwise comparisons and means in the same row without superscripts or that share the same superscript do not differ.

**Table 3:** Correlations of emotion dynamics across assessment methods.

Emotion dynamic	Correlation coefficients			Difference between correlations	
	EMA^a^-EOD^b^ diary	EMA-DRM^c^	EOD diary-DRM	χ^2^ (*df*=2)	*P* value
PA^d^ mean level	0.95^e^	0.80^f^	0.79^f^	50.52	<.001
NA^g^ mean level	0.95^e^	0.78^f^	0.74^f^	70.68	<.001
PA variability	0.68^e^	0.54	0.38^f^	15.46	<.001
NA variability	0.80^e^	0.47^f^	0.45^f^	25.68	<.001
PA instability	0.51^e^	0.33	.019^f^	9.05	.01
NA instability	0.70^e^	0.37^f^	0.41^f^	14.99	<.001
PA inertia	0.46^e^	0.18	.017^f^	6.43	.04
NA inertia	0.17	0.26	0.03	3.33	.19
Network density	0.36	0.32	0.26	0.77	.68
Mixed emotions	0.92^e^	0.76^f^	0.76^f^	30.35	<.001
Dialecticism	0.57^e^	0.17^f^	0.31	14.25	<.001

^a^EMA: ecological momentary assessment.

^b^EOD: end-of-day.

^c^DRM: day reconstruction method.

^d^PA: positive affect.

^e,f^Correlations in the same row with different superscripts differ significantly from each other using Bonferroni-adjusted pairwise comparisons and means in the same row without superscripts or that share the same superscript do not differ. Correlations >0.21 are significant at *P*<.05, correlations >0.28 are significant at *P*<01, and correlations >0.35 are significant at *P*<.001.

^g^NA: negative affect.

The goal of the primary analyses was to examine the comparability of measures that were computed in the way they would most likely be computed in applied research, that is, based on all available data for each ILA method. However, given that the DRM captures only 1 single day, an interesting question is whether the correspondence between EMA-based and DRM-based measures increases when measures of emotion dynamics are derived from EMAs for the exact day before the DRM was completed (ie, the exact day respondents were asked to rate in the DRM). Secondary analyses were conducted to address this question (based on 71 participants who had at least 3 EMA reports for the day before the DRM). The pattern of correlations with DRM-based measures was highly similar when measures of emotion dynamics were derived from all EMA data versus same-day EMA data (the median Cohen *q* for the difference in correlations was −0.014).

### Patterns of Associations Among Emotion Dynamics Within Each ILA Method

**Figure 1:**
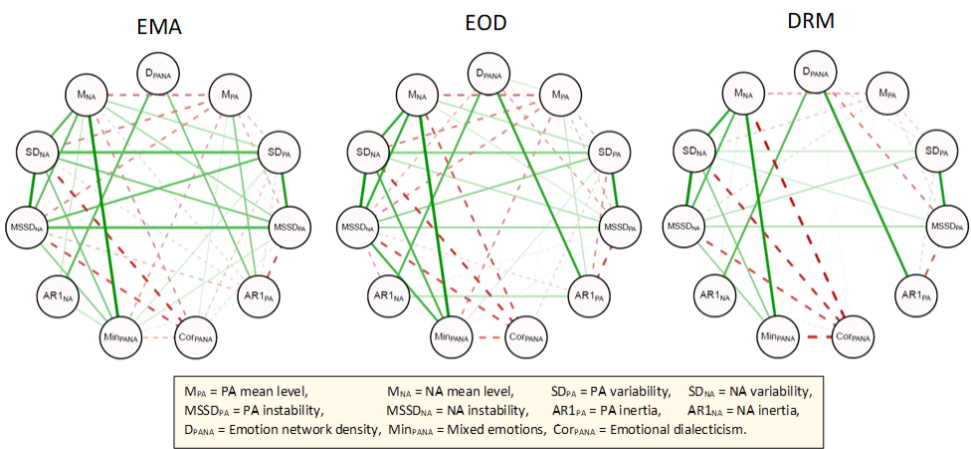
The network of pairwise correlations among emotion dynamics for each assessment method. Green lines represent positive relations, and red dashed lines represent negative relations. The thickness and transparency of the lines correspond with the magnitude of correlations. For clarity, lines are shown only for correlations that surpass the *P*<.05 significance threshold (above an absolute value of 0.21). EMA: ecological momentary assessment; EOD: end-of-day; DRM: day reconstruction method; PA: positive affect; NA: negative affect.

The network of pairwise associations among the various emotion dynamics is illustrated in Figure 1 for each ILA method. Examining the network of EMA-derived measures, several patterns are noteworthy. First, moderate-to-strong positive connections were evident among all variability and instability measures (ρ≥0.67, indicated by thick green lines). Second, the measure of mean NA levels showed positive connections with NA variability and NA instability and mixed emotions (ρ≥0.67). Third, although measures of mean PA levels and emotional dialecticism were weakly correlated with each other (ρ=0.33), both showed moderate-to-strong negative connections with mean NA levels, NA variability, and NA instability (ρ≤−0.54, indicated by thick red lines). Finally, emotion network density was positively associated with PA inertia and NA inertia (ρ≥0.59), whereas all 3 measures showed otherwise few connections in the network. Visual comparison of the correlation networks suggests that the patterns of association were similar for measures derived from EMAs and EOD diaries. However, DRM-derived measures generally showed fewer and weaker interconnections (Figure 1).

**Figure 2:**
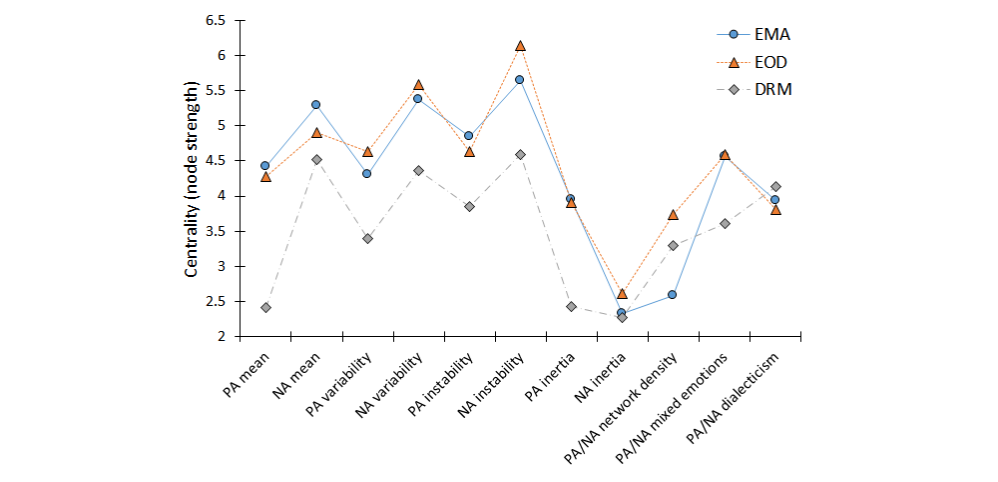
Centrality (node strength) of emotion dynamics for each assessment method. EMA: ecological momentary assessment; EOD: end-of-day; DRM: day reconstruction method; PA: positive affect; NA: negative affect.

The magnitude of the interconnections is quantified in the node strength centralities of the measures (Figure 2). For emotion measures derived from EMA, those tapping emotion levels, variability, and instability occupied the most central places with the strongest interconnections; measures for NA showed consistently greater centralities than corresponding measures for PA. As can be seen in Figure 2, the ordering of node strengths corresponded very closely between EMA measures and those derived from EOD diaries, with a correlation of ρ=0.92 between these 2 ILA methods. The node strength centralities of DRM-derived measures were generally lower (corresponding with weaker interconnections among DRM-derived measures), and the ordering of node strengths showed moderate-to-high correspondence with those from EMAs (ρ=0.75) and EOD diaries (ρ=0.70).

### Relationships With Health Outcomes

**Figure 3:**
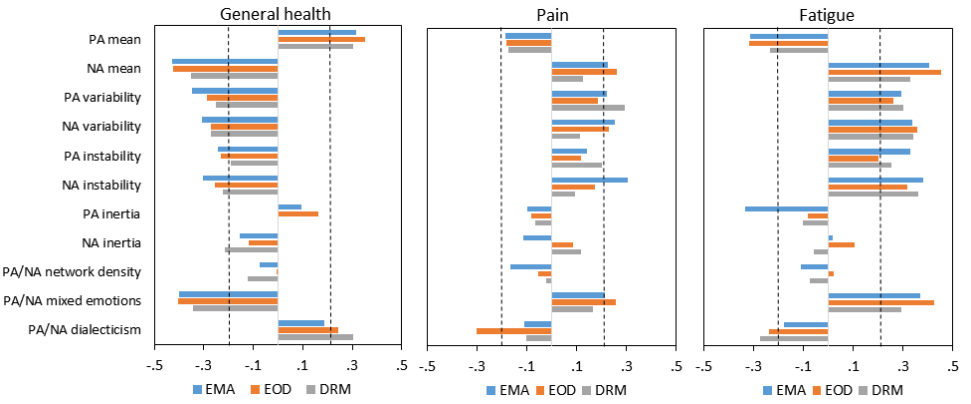
Correlations of measures of emotion dynamics with self-reports of general health, pain, and fatigue for each assessment method. Horizontal bars represent the magnitude and direction of correlation coefficients. Correlations exceeding a value of 0.21 (indicated by vertical dashed lines) are significant at *P*<.05. EMA: ecological momentary assessment; EOD: end-of-day; DRM: day reconstruction method; PA: positive affect; NA: negative affect.

Finally, relationships between the measures of emotion dynamics and health outcome variables were examined. Overall, the emotion dynamics showed small- to medium-sized correlations with self-reports of general health and fatigue, and somewhat less pronounced correlations with pain (Figure 3). Comparisons of the correlation coefficients between ILA methods yielded very few differences: out of 33 Wald tests (for 11 emotion dynamics and 3 health outcomes), only 1 indicated a significant difference between the ILA methods (Wald χ^2^_2_=6.19, *P*=.04 for the correlations between PA inertia and fatigue; post hoc pairwise comparisons showed that the correlation was significantly more negative for EMA than for EOD diary reports, z=−2.38, *P*=.02). Average effect sizes (absolute values of Cohen *q*) for the differences in correlations between the ILA methods were very small for EMAs versus EOD diaries (general health, mean absolute *q*=0.04; pain, mean absolute *q*=0.08; and fatigue, mean absolute *q*=0.09), EMAs versus DRM (general health, mean absolute *q*=0.07; pain, mean absolute *q*=0.10; and fatigue mean absolute *q*=0.08), and for EOD diaries versus DRM (general health, mean absolute *q*=0.07; pain, mean absolute *q*=0.09; and fatigue, mean absolute *q*=0.08).

As shown in Figure 3, for all ILA methods, the most pronounced relationships with health outcomes were evident for mean emotion levels, variability, and instability measures in expected directions. Higher mean PA levels were consistently correlated with better general health, less pain, and less fatigue, whereas higher mean NA, variability (PA and NA), and instability (PA and NA) correlated with poorer general health, more pain, and more fatigue. Higher values of emotional dialecticism were associated with better health outcomes, whereas, contrary to theoretical expectations [37], more mixed emotions were associated with worse health outcomes. Finally, inertia (PA and NA) and emotion network density measures showed almost no relationship with the health outcomes.

We also explored whether measures of emotion dynamics demonstrated incremental validity in predicting the health outcomes above and beyond average emotion levels. To examine this, a series of multiple regression analyses were conducted in which the health outcomes were regressed on a measure of emotion dynamics after controlling for PA and NA mean levels separately for each ILA method. In these models, the only significant predictor of general health was the mean level of NA, consistently for each ILA method (*P*<.05 in all instances). For pain as an outcome variable, no emotion measure (including mean PA and NA) was a significant predictor in the multiple regressions; the exception was that PA variability uniquely predicted pain in the DRM (*P*=.02). For fatigue, the only significant predictor was the mean level of NA, again consistently for each ILA method (*P*<.05 in all instances). In addition, PA variability uniquely predicted fatigue in the DRM (*P*=.03).

## Discussion

Emotions fluctuate and change over time, and ILA methods are uniquely suited to quantify the temporal dynamics of people’s emotional lives. There has been a surge of interest in creating measures that tap a variety of emotion dynamics from ILA. To our knowledge, this is the first study to directly compare EMA, EOD diary, and DRM ILA methods in the measurement of intraindividual emotion dynamics. If different ILA methods produce noncorresponding measures of emotion dynamics, this would have important research implications in that it would question the validity of the measures and threaten the reproducibility of empirical research results.

EMAs and EOD diaries are arguably the 2 most commonly used ILA methods [4]. Previous research has documented that individual differences in mean levels of positive and negative experiences are highly correlated between these methods [38,39]. This finding was confirmed in this study. Expanding on previous research, we found that several measures of emotion dynamics derived from EOD diaries also reproduced those derived from EMAs very well. Specifically, measures of emotion variability, instability, mixed emotions, and emotional dialecticism showed substantial correspondence between the 2 methods, with comparable mean levels and moderate-to-high correlations. Considering the argument that momentary and daily fluctuations in emotions may be quite different conceptually, the level of agreement between the methods is perhaps somewhat surprising. For example, given that EMAs encompass the sum of within-day and between-day sources of intraindividual variation, whereas EOD diary ratings are limited to between-day variation, one might expect the average variability to be higher in EMAs than in EOD diaries, which we did not find. Our results suggest that differences in the time scale and frequency of measurement inherent in EMA and EOD diary ratings do not dramatically impact measures of emotion variability, instability, mixed emotions, and emotional dialecticism.

However, this does not mean that EMA and EOD diary measures can be viewed as universally interchangeable. Measures of inertia and emotion network density were correlated at levels below 0.50 between the methods. In contrast to the other measures of emotion dynamics, inertia and emotion network density measures are specifically focused on temporal dependencies between successive self-report ratings; that is, they capture the rate (or speed) of changes rather than the magnitude of changes. This suggests that the measurement of how emotions evolve over time and at what rate may not be captured in the same way by EOD diaries and EMAs.

The DRM was originally developed as an alternative to EMAs for use in large and population-representative samples, yet few previous studies have directly compared the information obtained from the DRM with that obtained from EMAs [40-42] or EOD diaries [43,44]. In this study, the DRM demonstrated high correspondence with other ILA methods for measures of average emotion levels and mixed emotions and moderate correspondence for emotion variability. For more complex emotion dynamics, the DRM showed low correlations with EMAs and EOD diaries. Similarly, the networks of pairwise associations among the various emotion dynamics were only moderately concordant between the DRM and the other ILA methods. This suggests that the DRM may adequately capture people’s average emotion levels (and, to some extent, their emotion variability) but may not serve as a direct replacement of other ILA methods in research on more intricate emotion dynamics.

Interestingly, all ILA methods produced very similar patterns of relationships with health outcomes. Higher mean PA and lower mean NA levels were associated with better physical health outcomes with medium (general health and fatigue) and small-to-medium (pain) effect sizes, replicating previous research on these mind-body relationships [45-47]. The consistency of these results across ILA methods suggests that each of them can equally contribute to understanding the linkages between people’s general emotion levels and health outcomes in older adulthood.

Measures of emotion variability and more emotional instability derived from each ILA method were also consistently related to health outcomes in expected directions. These results are noteworthy considering that even though the last decades have witnessed a surge in research linking emotion variability with maladaptive outcomes, most of this research has focused on psychological well-being (eg, depression, anxiety) rather than physical health outcomes. In a large meta-analysis, Houben et al [9] found small-to-medium effect sizes for relationships between psychological well-being and measures of emotion variability (ρ=0.18) and instability (ρ=0.21). Our results suggest effect sizes of similar magnitude for physical health outcomes.

Measures of mixed emotions (MIN index) and emotional dialecticism (within-person correlation of PA and NA) were moderately negatively correlated with each other and showed opposite relationships with the health outcomes within each of the ILA methods. This finding would appear counterintuitive, given that the 2 measures aim at capturing conceptually similar concepts. However, previous studies have found a generally weak correspondence between these indices of mixed emotions and emotional dialecticism [13,26], suggesting that they measure different aspects of emotional experience. Consistent with our results, higher emotional dialecticism scores have previously been shown to predict fewer health symptoms [12], whereas mixed emotions assessed with the MIN index were associated with more physical disability across adulthood [13].

Measures that focus on temporal dependencies of emotional states (emotional inertia and emotion network density) were practically uncorrelated with the physical health outcomes in this study, contrary to previous meta-analytic findings that higher emotional inertia relates to worse psychological well-being, albeit with overall small effect sizes [9]. It is possible that temporal dependency measures play a lesser role in understanding physical health compared with mental health. However, previous simulation studies have also demonstrated that measures of inertia derived from ILAs tend to have very low reliability, and this may have attenuated the observed correlations with health outcomes. A recent Monte Carlo simulation by Du and Wang [17] suggests that even if the emotional states themselves are assessed with near-perfect reliability, up to 100 measurement occasions per person may be necessary to obtain reliabilities >0.70 for inertia measures, whereas individual differences in mean levels, variability, and instability measurement required substantially fewer measurement occasions to be reliably measured in the simulation study. Future research would benefit from implementing ILA methods across multiple waves (eg, using measurement bursts) as a means to estimate the test-retest reliability of measures of emotion dynamics and to correct their correlations with health outcomes for unreliability in empirical samples.

It is also noteworthy that once the explanatory power of mean levels of PA and NA was taken into account, measures of emotion dynamics showed little to no added value in the prediction of the health outcomes, regardless of the ILA method. This finding corresponds with recent findings by Dejonckheere et al [21], suggesting that more complex emotion dynamics add little to the prediction of psychological well-being and emotion disorders after controlling for mean affect levels. Our findings suggest that caution is similarly warranted when assuming that measures of complex emotion dynamics have unique predictive utility for understanding physical health parameters, even though this would need to be confirmed in larger studies.

Several limitations of this study need to be considered. First, the study was restricted to older individuals aged 50 years and above, and the results may not be generalizable to younger adults. However, evidence for convergent validity across ILA methods in this age group may be particularly important given that older people may have more problems with electronic ILA data collection and that potential cognitive problems in this age group may interfere with accurate self-reports [48]. Second, the sample size of 90 respondents was relatively modest, although it was comparable with previous studies examining the correspondence of EMAs with EOD diaries [38] or the DRM [40,41]. A third limitation is that whereas EMAs and EOD diaries used the same wording of affect items (ie, happy, dejected/blue/downhearted) and the same response scale (a 0-100 visual analog scale), the DRM used a partially different wording (ie, happy, sad) and different response scale (a 7-point numeric response scale) to collect affect ratings. This may have artificially deflated the correspondence between measures of emotion dynamics derived from the DRM and the other ILA methods. Holding the items and response scales constant across ILA methods in future studies would enhance the rigor of comparisons by minimizing the potential impact of such extraneous factors. Fourth, it is also possible that completing multiple EMA ratings throughout the day impacted participants’ EOD diary and DRM affect ratings, which may have artificially inflated the correspondence between the different measures. Previous literature found little evidence that recall ratings are impacted by momentary reporting [49], but we did not specifically examine this possibility here. Finally, even though we examined a variety of measures of emotion dynamics, the study was limited to measures that can be derived from 2 affect questions. Additional measures of emotion dynamics that have been proposed in the literature were not considered here because they require administration of multiple PA or NA items at each measurement occasion (examples are emotional granularity, which captures the extent to which individuals differentiate between multiple emotions of the same valence [50], and emodiversity, which captures the extent to which individuals experience a narrow or wide range of different emotions [51]).

In summary, EMAs and EOD diaries correspond moderately to highly with each other in the information they provide about individual differences in various emotion dynamics. Compared with these ILA methods, the DRM provides corresponding information about emotion levels and (to a lesser extent) variability, but not about more complex emotion dynamics. Our results caution researchers against viewing these ILA methods as universally interchangeable. Although measures of emotion dynamics derived from all ILA methods showed small-to-moderate relationships with physical health outcomes, the unique predictive ability of more complex emotion dynamics for understanding health outcomes remains to be established.

## Abbreviations

ANOVA: analysis of variance
DRM: day reconstruction method
EMA: ecological momentary assessment
EOD: end-of-day
ILA: intensive longitudinal assessment
NA: negative affect
PA: positive affect
UAS: Understanding America Study
USC: University of Southern California
